# Type 1 Diabetes and Osteoporosis: From Molecular Pathways to Bone Phenotype

**DOI:** 10.1155/2015/174186

**Published:** 2015-03-22

**Authors:** Tayyab S. Khan, Lisa-Ann Fraser

**Affiliations:** ^1^Department of Medicine, Western University, London, ON, Canada; ^2^Division of Endocrinology and Metabolism, St. Joseph's Health Care, 268 Grosvenor Street, London, ON, Canada N6A 4V2

## Abstract

The link between type 1 diabetes mellitus (DM1) and osteoporosis, identified decades ago, has gained attention in recent years. While a number of cellular mechanisms have been postulated to mediate this association, it is now established that defects in osteoblast differentiation and activity are the main culprits underlying bone fragility in DM1. Other contributing factors include an accumulation of advanced glycation end products (AGEs) and the development of diabetes complications (such as neuropathy and hypoglycemia), which cause further decline in bone mineral density (BMD), worsening geometric properties within bone, and increased fall risk. As a result, patients with DM1 have a 6.9-fold increased incidence of hip fracture compared to controls. Despite this increased fracture risk, bone fragility remains an underappreciated complication of DM1 and is not addressed in most diabetes guidelines. There is also a lack of data regarding the efficacy of therapeutic strategies to treat osteoporosis in this patient population. Together, our current understanding of bone fragility in DM1 calls for an update of diabetes guidelines, better screening tools, and further research into the use of therapeutic strategies in this patient population.

## 1. Introduction

Osteoporosis is the most common bone disease known, affecting an estimated 200 million people worldwide [1]. Approximately 30% of all postmenopausal women are affected and up to 40% will develop a fragility fracture within their lifetime [2]. Over time, a number of risk factors have been associated with osteoporosis and are useful when used in screening tools and treatment algorithms [3–7]. Diabetes, although identified more than half a century ago as being associated with bone frailty [8], has come to the forefront only within the last decade as an important osteoporosis risk factor.

While both type 1 (DM1) and type 2 (DM2) diabetes have been found to increase fracture risk, the link is far more profound with DM1. DM1 accounts for approximately 5% of all cases of diabetes in the USA [9] and is associated with a 6.4–6.9-fold increase in the relative risk of hip fracture compared to individuals without diabetes [10, 11]. As life expectancy continues to increase for those living with DM1 [12], a further increase in the number of fractures occurring in this population is expected in the future.

Although we are now beginning to understand some of the genetic and molecular pathways underlying the association between DM1 and bone fragility, many questions remain. Answers to these questions will be critical to further our understanding of osteoporosis in this patient population and key to developing effective screening tools and treatment options.

This review explores the current understanding of cellular and molecular pathways underlying the bone fragility found in individuals with DM1. The impact of these factors on bone quality and fracture risk is described, and avenues for future research, which could help further the development of new therapeutic modalities for this patient population, are explored.

## 2. Pathophysiology of Bone Fragility in Type 1 Diabetes

Bone remodelling is dependent on a precise balance between bone formation, carried out by osteoblasts, and bone resorption, caused by osteoclasts. This balance is regulated by a myriad of molecular signals. Disruptions in any of these molecular pathways can disturb the equilibrium of bone turnover and thereby affect bone quality. Multiple changes in cellular, microarchitectural, and humoral factors involved in bone remodelling have been identified in the setting of DM1.

### 2.1. Osteoblasts (Bone Formation)

Impaired osteoblast activity and differentiation are central to the bone fragility seen in patients with DM1. Initial clues pointing to this were identified in pediatric studies which consistently demonstrated a decrease in bone formation marker carboxyterminal propeptide of type 1 collagen (PICP) among children with DM1 [13, 14]. Studies in rodent models provided further insights into the molecular basis underlying this defect and suggested that DM1 not only affects osteoblast function, but also modulates multiple steps along the osteoblast differentiation pathway in the bone marrow. Studies using the streptozotocin induced rat model of DM1 showed that this differentiation defect starts at the stem cell level, with reductions in both the total number of mesenchymal stem cells as well as osteoblast progenitor cells. These changes were found to be, at least partly, mediated by an increase in apoptosis of the mesenchymal stem cells [15, 16]. Recent studies reinforce these findings and show that embryonic stem cells (ESCs) grown in high concentrations of glucose possess reduced potential for self-renewal as shown by a decrease in the number of colonies of undifferentiated cells compared to stem cells grown in physiologic glucose concentrations [17]. This suggests that part of the osteoblast deficit among DM1 is mediated by a failure to maintain pluripotent stem cells for osteoblast differentiation.

Several studies have identified the genetic players mediating impaired osteoblast differentiation in DM1. This was demonstrated by a marrow ablation study in streptozotocin induced DM1 mice which examined markers of osteoblast differentiation, including runt domain factor-2 (Runx-2) and drosophila distal less gene (Dlx-5). This study demonstrated a decrease in both Runx-2 and Dlx-5 among DM1 mice, compared to nondiabetic controls, a defect rescued to control levels by treatment with insulin [18]. Reductions were also seen in the expression of osteocalcin [18], which is expressed downstream of Runx-2 [19] and not only regulates bone homeostasis, but also is an important mediator in the interaction between bone and glucose metabolism, as it promotes proliferation of pancreatic beta cells and stimulates insulin release [20]. Indeed there is evidence that this reduction in osteocalcin, as well as other markers of bone formation, including alkaline phosphatase and collagen synthesis, may at least partly be mediated by lack of insulin which has been shown to directly stimulate activity of these markers in osteoblasts* in vitro* [21]. These results were complemented by a later study in streptozotocin induced rats which not only showed reductions in Dlx-5, Runx-2, and osteocalcin levels, but also an increase in sclerostin (Scl) and Dickkopf-1 (Dkk1) levels, both of which are negative regulators of Wnt signalling which is essential for osteoblast differentiation [22]. Taken together, these findings offer interesting insights into the mediators of bone frailty in individuals with DM1 and also provide important clues to the interactions between bone cells and glucose metabolism.

### 2.2. Osteocytes

Osteocytes comprise greater than 90–95% of all bone cells, are the principle mechanosensors of bone, and, through an extensive network of canaliculi, closely regulate bone remodelling [23]. These cells have been found to play an important role in modulating the bone fragility in DM1. Early histomorphometric analyses identified a reduction in markers of osteocyte activity including a reduction in total lacunar density and osteocyte density in a rat model of DM1 [24]. These results were replicated in a study that demonstrated a reduction in osteocyte number, at least partly due to increased apoptosis, in mice with DM1 compared to nondiabetic controls [25]. As osteocytes exclusively secrete sclerostin, the negative regulator of Wnt signalling pathway essential for osteoblast differentiation, it has been postulated that sclerostin may have a key role in shaping bone quality in DM1. Unlike reports of increased sclerostin activity inhibiting the Wnt pathway in DM1 rats [22], a murine study showed a decrease in its activity in DM1 mice [25]. Whether this was due to interspecies variability, or variations in assays used, is unclear. Despite this, Wnt activity was reduced in both studies. In humans, data from a recent cross-sectional study showed increased sclerostin levels among individuals with DM2, with only a trend towards an increase among younger, but not older, patients with DM1, compared to nondiabetic controls [26]. More recent studies, however, have suggested an increase in sclerostin levels among both males and females with longstanding DM1 (22.4 ± 9.5 years) compared to controls [27], with another study also demonstrating longer duration of DM1 (>15 years) with higher sclerostin levels [28]. Collectively, these findings suggest that DM1 may affect the Wnt pathway and subsequently osteoblast differentiation, at multiple levels including osteocyte-osteoblast interaction; further studies are needed to elucidate the role of sclerostin in modulating these effects.

### 2.3. Osteoclasts (Bone Resorption)

Unlike the effects of DM1 on osteoblasts, the results of studies examining the role of DM1 in mediating osteoclast function have been mixed.* In vitro* studies displayed decreased osteoclast activity as measured by tartrate resistant alkaline phosphatase (TRAP) in embryonic stem cells grown in hyperglycemic versus physiologic culture media [17]. In contrast, osteoclasts from DM1 mice, despite their smaller size, demonstrated increased bone resorption in response to addition of receptor activator of nuclear factor kappa-B ligand (RANK-L) and macrophage colony stimulating factor (M-CSF) [29]. Levels of osteoprotegerin (OPG), a soluble receptor of RANK-L, which negatively regulates osteoclast differentiation, were found to be reduced in animal models of DM1, leading to increased bone resorption [30]. Interestingly, this increased osteoclast activity was not seen in animal studies where untreated DM1 rats actually had a reduced number of osteoclasts at the femoral neck region compared to nondiabetic controls, a finding correlated with decreased bone quality at this site in these rats [31]. This suggests that bone fragility in these animal models may either be principally mediated by decreased osteoblast activity or be secondary to the continued accumulation of bone microdamage due to reduced osteoclast activity. These results have been replicated in other animal studies showing no change, or even reduction, in bone resorption following induction of diabetes [22, 32, 33]. Finally, results of human studies evaluating the effects of diabetes on markers of bone resorption have been mixed, with both an increase [34] and a decrease [35] reported in different studies among individuals with DM1. Whether these differences are due to variability in patients' age, markers used, or a true representation of physiology is unclear.

Given the consistent data regarding impaired osteoblast differentiation and function in DM1, it appears that osteoblasts take a leading role in determining the bone phenotype in this patient population. The bone fragility found in DM1 seems to be largely caused by a decrease in bone formation. It is possible that any derangements in bone resorption (osteoclast function) may be, at least partly, mediated by signalling from osteoblasts to osteoclasts.

### 2.4. Bone Matrix

Besides having direct effects on the cells involved in bone remodelling, DM1 also affects the bone matrix, thereby modulating bone quality. These effects are mediated by the formation of advanced glycation end (AGEs) products, which are produced due to nonenzymatic glycosylation of proteins or lipids and are implicated in multiple diabetes complications, including bone fragility [36]. Evidence for this was provided by a study in rats with DM1 which showed an increase in the nonenzymatic cross-linking in diabetic rats compared to controls [37]. Complementing these results are those from a clinical cross-sectional study that demonstrated that higher serum levels of pentosidine, an AGE product, were associated with fractures in patients with DM1 after adjusting for age, BMI, family history of fractures, smoking, vitamin D deficiency, BMD at lumbar spine, femoral neck, and total hip [38]. While enzymatic cross-linking is beneficial, the nonenzymatic AGEs confer inferior bone quality [39]. First, as both enzymatic and nonenzymatic cross-links happen on lysine residues, formation of AGEs competitively inhibits the site(s) left for enzymatic cross-linking [40]. Secondly, AGEs can cause osteoblast apoptosis through activation of the receptor for advanced glycation end products (RAGE) [41], suggesting that some effects of diabetes on osteoblast cell apoptosis may be mediated by AGEs.

### 2.5. Diabetic Complications

In addition to the effects of DM1 on bone cells and the bone matrix directly, diabetic complications occurring in other body systems are important in influencing fracture risk. Ocular, cardiovascular, neurologic, and renal complications resulting from diabetes are associated with a greater than tenfold increase in the relative risk of fracture among both men and women [10, 42]. While ocular, cardiovascular, and neurologic complications, as well as hypoglycemic episodes, may increase fracture risk by increasing the risk of falls, the effects of nephropathy may be mediated by derangements in vitamin D and subsequently parathyroid hormone metabolism [10].

## 3. Bone Phenotype in Type 1 Diabetes

The cellular and molecular mechanisms described above converge to affect several parameters of bone quality, culminating in a higher risk of fracture among individuals with type 1 diabetes.

### 3.1. Bone Mineral Density

Among the site(s) at risk for osteoporotic fractures, the bone site that is most consistently shown in studies to be associated with reduced bone mineral density (BMD) in DM1 is the hip. While some exceptions exist [43], most studies demonstrate inferior hip BMD among those with DM1 compared to controls without diabetes. In a meta-analysis combining results of five studies, Vestergaard [10] demonstrated a significant reduction in *Z* scores at the hip (*Z* score: −0.37 ± 0.16, *P* < 0.05) among patients with DM1 compared to controls. Findings from a case control study by Eller-Vainicher et al. [44] were similar, where a reduction in femoral neck BMD *Z* scores was seen among patients with DM1 (−0.32 ± 0.14) compared to controls (0.63 ± 1.0, *P* < 0.0001) matched for age, BMI, and gonadal function. Similarly, a prospective study has identified a decrease in femoral neck BMD among males with DM1; however, the association was not replicated in females in this study [45]. Other studies however, looking specifically at females, have identified the association between decreased BMD and DM1, with one study showing reduced femoral shaft BMD to be associated with the age of onset of DM1 [46].

Similar to trends seen at the hip, most studies evaluating BMD at the lumbar spine have identified lower *Z* scores in those with DM1 compared to controls (e.g., −1.03 + 1.19 versus −0.57 + 1.04, *P* < 0.01 [45]; −0.55 ± 1.3 versus 0.35 ± 1.0, *P* < 0.0001 [47]). The exact onset of these alterations in BMD is unclear. Studies of adolescents with DM1 have shown conflicting results, with some studies showing a decrease in lumbar spine BMD [13, 14, 48] and other studies showing no difference [43, 49]. Despite this, factors underlying this defect in lumbar spine BMD are beginning to be identified, as demonstrated by multiple studies showing an inverse correlation between BMD *Z* scores and glycosylated hemoglobin (HgbA1C) [44, 50, 51], as well as the identification of an association between lower lumbar spine BMD and the duration of diabetes [13].

Unlike the decline in BMD seen at the hip and spine, data regarding BMD at the wrist in individuals with DM1 have been conflicting. A study of young patients with DM1 revealed no significant differences in volumetric trabecular BMD measured using quantitative computed tomography (qCT) at the wrist compared to healthy controls [52]. Similarly, another study found no decrease in areal BMD at the forearm in either males or females with DM1 compared to controls [53]. However, a different study in patients with long standing DM1 (mean duration 16 years) demonstrated that a higher proportion of patients with DM1 (48%; *P* < 0.05) had forearm BMD *Z* scores ≤ 1 compared to controls (28%) [54]. Therefore it is possible that a decline in forearm BMD may be something that is only seen after longstanding diabetes.

### 3.2. Bone Geometry

In addition to its effects on BMD, type 1 diabetes also modulates fracture risk by affecting bone geometry, which, along with microarchitecture, modulates the structural properties of bone. Bone geometry includes the size and shape of a bone and is an important determinant of bone fragility. Studies done at multiple sites in human cadavers have demonstrated larger bones to have greater strength [55]. In addition, geometry also includes the distribution of mass in a bone which determines its resistance to bending and torsional load [56]. A study in adolescents with DM1 showed reduced whole body and lumbar spine bone area, measured using dual energy X-ray absorptiometry (DEXA), and reductions in both cortical and trabecular bone areas at the tibia, measured using peripheral quantitative computed tomography (pQCT) [57]. A later study controlled for gender differences by comparing male and female adolescents with DM1 to gender matched nondiabetic controls found no differences in any of the geometric parameters tested, at the proximal femur, lumbar spine, distal radius, distal tibia, and tibial shaft for either gender, except for a significantly reduced cortical cross-sectional area (86.8 mm^2^ versus 98.6 mm^2^, *P* < 0.01) among males with DM1 at the radial shaft [58]. The authors concluded that this may be showing gender and site specific regulation of bone geometry in DM1. Whether these differences persist longitudinally is unclear. In fact, in a longitudinal evaluation of young patients with DM1, the reductions in radial cortical, trabecular, and total surface area among patients with diabetes seen at baseline normalized, to levels comparable to controls, at a second measurement 5.5 years later [59]. Whether the normalization of this geometric defect is site-specific remains to be established and further longitudinal studies are needed to establish if this could be generalized to other anatomic skeletal sites.

### 3.3. Bone Mechanics and Fracture Risk

While data gathered from various indices of bone quality can predict fracture risk, it is well accepted that none of these parameters can fully explain bone mechanical properties, which in turn serve as the gold standard for determining the mechanical competence of a bone and its resistance to fractures [60]. Bone mechanical properties are dependent on their structural (dependent on bone geometry and microarchitecture) and material (influenced by intrinsic bone properties including mineralization and collagen integrity) properties. There is an absence of clinical, or cadaveric, studies specifically examining the mechanical properties of bone strength in those with DM1. Limited data, however, from animal models suggests that both structural and material bone mechanical properties are compromised in rat models of DM1 [61, 62]. It is unclear if this loss of strength can be avoided by improving glycemic control, as results from studies have been conflicting. One study demonstrated a reversal of the biomechanical defect in bone in rats following maintenance of good glycemic control [61], but the same effect was not replicated in another study [62]. Despite this, both studies demonstrated deterioration in both structural and material properties in rats with DM1, providing evidence for the multifactorial effects of the disease on bone phenotype.

Many studies to date have shown an increased risk of fracture in patients with DM1. As mentioned above, the majority of the data that is available is for hip fractures, for which a large meta-analysis demonstrated that, in the setting of DM1, the relative risk of 6.94 (95% C.I. 3.25–14.78, *P* < 0.01) was much higher compared to 1.42 as predicted by BMD measurement alone [10]. These results confirm that the increase in fracture risk associated with DM1 is multifactorial, not simply a result of a decline in BMD.

While multiple large studies evaluating the risk of vertebral fracture in DM1 are not available to be able to undertake a meta-analysis, there is data to suggest an increase in fracture risk at this site. This was seen in a cross-sectional case cohort study involving patients of both genders with DM1, who demonstrated a higher prevalence of fractures compared to controls (24.4 versus 6.1%, *P* = 0.002) [47]. A population based, case cohort study showed similar findings, with patients with DM1 displaying a higher risk of spinal fractures (OR 2.5, 95% C.I. 1.3–4.6) compared to nondiabetic controls [63], providing evidence that DM1 modulates fracture risk at multiple anatomic sites.

## 4. Future Directions

DM1 affects bone phenotype via multiple pathways, leading to a significantly increased risk of hip and spine fracture and subsequent morbidity and mortality. This underlines the need for active screening and, if warranted, treating this patient population so as to reduce this risk. It is therefore not surprising that multiple osteoporosis guidelines, including the Osteoporosis Canada 2010 guidelines, list DM1 as a risk factor for osteoporosis and fractures and call for [64], or imply [65], earlier bone screening among these individuals. However, similar recommendations to suggest osteoporosis screening among patients with diabetes, particularly DM1, are not found in the most recent Canadian Diabetes Association Clinical Practice Guidelines [66]. While guidelines from the American Diabetes Association [67] do mention osteoporosis as a comorbid condition with diabetes, multiple European diabetes guidelines do not mention osteoporosis as a complication of or an associated condition with DM1 [68, 69]. Hence, there is a lack of consensus amongst professional societies as to the importance and significance of osteoporosis and fractures in the DM1 population.

As mentioned above, the bone fragility associated with DM1 is principally mediated by a decrease in osteoblast activity, which in turn is related to defects in the number, self-renewal, and differentiation of pluripotent mesenchymal stem cells. There is a growing body of literature linking osteoporosis to stem cell defects which have been identified in both animal and human studies [70–73]. There is also evidence suggesting that the effects of medications used to treat osteoporosis, including bisphophonates, may be partially mediated by increasing the number, or osteogenic potential, of mesenchymal stem cells [74–76]. Collectively, these studies suggest that further research into therapies aimed at increasing the pool of pluripotent mesenchymal stem cells, or increasing their potential to form osteoblasts, could hold promise in the treatment of osteoporosis in general and particularly among individuals with DM1.

While bisphosphonates principally work by suppressing osteoclast induced bone resorption [77], they have been used for treatment of osteoporosis in patients with DM1 based on extrapolation from a prospective study in a subgroup of patients with DM2 from the Fracture Intervention Trial (FIT). In this study, treatment with alendronate lead to a significant increase in hip and spine BMD from baseline in patients with DM2 (+2.4 ± 0.4%, *P* < 0.001 at hip; +6.6 ± 0.5%, *P* < 0.001 at lumbar spine) compared to placebo (0.9 ± 0.4% at the hip, −1.9 ± 0.4% at the lumbar spine) [78]. Complementing this is a retrospective cohort study which demonstrated no difference in antifracture efficacy of bisphosphonates between patients with and without diabetes and also between patients with DM1 versus DM2 [79]. Despite this, there is evidence that bone turnover, even at baseline, may be suppressed in individuals with DM1 [35], which may add to fracture risk in these patients. Further suppression of bone turnover, at least theoretically, could increase microdamage and consequently increase the risk of atypical fractures in these individuals. Indeed, the proportion of patients with diabetes (both types 1 and 2) among postmenopausal women who suffer an atypical femoral fracture is more than twice that of those without diabetes (11.6% versus 5.6%) [80]. This information, coupled with the pathophysiology of the bone phenotype in DM1, calls for a prospective analysis of the long term safety of bisphosphonate therapy in this patient population.

Given the above theoretical concerns about the use of bisphosphonates and other antiresorptive therapies, bone anabolic therapies, including teriparatide, seem to hold promise as ideal treatment options for osteoporosis in DM1. Initial results from animal studies show that intermittent PTH is associated with an increase in trabecular BMD in mice with DM1, an increase mediated by suppression of osteoblast apoptosis, and improvements in mineral apposition and osteoblast surface [81]. These results were replicated in another murine study where administration of PTH related peptide (PTHrP) increased not only osteocyte number, but also Wnt signalling essential for osteoblast differentiation in DM1 mice [25]. Further research into the role of PTH for bone protection in patients with DM1 could hold interesting insights into its use as the therapeutic strategy of choice in this patient population.

Given the essential role of Wnt signalling in osteoblast differentiation and its inhibition by sclerostin described above, sclerostin is an attractive target for osteoporosis treatment, particularly in DM1. Indeed romosozumab, a monocloncal antibody against sclerostin, was well tolerated and caused increases in BMD at the lumbar spine (+11.3%, 95% C.I. 10.3–12.4, *P* < 0.001) and total hip (+4.1%, 95% C.I. 3.5–4.8, *P* < 0.001) compared to placebo in postmenopausal women in recent phase 2 clinical trials [82]. While the role of sclerostin antibody has been studied in animal models of DM2, where it leads to an increase in bone mass and mechanical properties [83, 84], similar studies in the setting of DM1 have not been done. These studies could be important in clarifying the effect of inhibition of sclerostin and its potential use against osteoporosis in DM1.

## 5. Conclusion

DM1 leads to increased bone fragility by modulating osteoblast differentiation and activity, causing derangements in osteocyte-osteoblast signalling and formation of AGEs in the bone matrix. Similarly, diabetic complications affect vitamin D metabolism and increase fall risk. The convergence of these factors leads to an inferior bone quality and an increased risk of fracture, particularly at the hip. Although this risk is acknowledged in osteoporosis guidelines, it remains underappreciated in most major diabetes guidelines, leading to a lack of awareness amongst clinicians caring for patients with DM1. There is also a lack of studies evaluating the efficacy of currently available, as well as emerging, therapeutic modalities to decrease fracture risk in individuals with DM1. Current diabetes guidelines need to be updated to recognise the link between bone fragility and DM1. Similarly, better screening tools need to be developed, and further research into therapies in this particular patient population is needed.
